# Variation in *Aedes aegypti* Mosquito Competence for Zika Virus Transmission

**DOI:** 10.3201/eid2304.161484

**Published:** 2017-04

**Authors:** Christopher M. Roundy, Sasha R. Azar, Shannan L. Rossi, Jing H. Huang, Grace Leal, Ruimei Yun, Ildefonso Fernandez-Salas, Christopher J. Vitek, Igor A.D. Paploski, Uriel Kitron, Guilherme S. Ribeiro, Kathryn A. Hanley, Scott C. Weaver, Nikos Vasilakis

**Affiliations:** University of Texas Medical Branch, Galveston, Texas, USA (C.M. Roundy, S.R. Azar, S.L. Rossi, J.H. Huang, G. Leal, R. Yun, S.C. Weaver, N. Vasilakis);; Centro Regional de Salud Pública, Tapachula, México (I. Fernandez-Salas);; University of Texas Rio Grande Valley, Brownsville, Texas, USA (C.J. Vitek);; Ministério da Saúde, Candeal, Salvador, Brazil (I.A.D. Paploski, G.S. Ribeiro);; Universidade Federal da Bahia, Salvador (I.A.D. Paploski, G.S. Ribeiro);; Emory University, Atlanta, Georgia, USA (U. Kitron);; New Mexico State University, Las Cruces, New Mexico, USA (K.A. Hanley)

**Keywords:** *Aedes aegypti*, Zika, transmission, vector competence, vector-borne infections, viruses, arbovirus, flaviviruses, mosquitoes, Zika virus

## Abstract

To test whether Zika virus has adapted for more efficient transmission by *Aedes aegypti* mosquitoes, leading to recent urban outbreaks, we fed mosquitoes from Brazil, the Dominican Republic, and the United States artificial blood meals containing 1 of 3 Zika virus strains (Senegal, Cambodia, Mexico) and monitored infection, dissemination, and virus in saliva. Contrary to our hypothesis, Cambodia and Mexica strains were less infectious than the Senegal strain. Only mosquitoes from the Dominican Republic transmitted the Cambodia and Mexica strains. However, blood meals from viremic mice were more infectious than artificial blood meals of comparable doses; the Cambodia strain was not transmitted by mosquitoes from Brazil after artificial blood meals, whereas 61% transmission occurred after a murine blood meal (saliva titers up to 4 log_10_ infectious units/collection). Although regional origins of vector populations and virus strain influence transmission efficiency, *Ae. aegypti* mosquitoes appear to be competent vectors of Zika virus in several regions of the Americas.

Zika virus is an emerging arthropodborne virus (arbovirus) of the family *Flaviviridae*. Discovered in 1947 (*1*), Zika virus remained obscure and its detection largely limited to sylvatic transmission cycles between arboreal mosquitoes (*Aedes* [*Stegomyia*] *africanus*, *Ae.* [*Diceromyia*] *furcifer*) and primates (*1*). Before the recent outbreaks in Micronesia (*1*) and French Polynesia (*2*), only 14 human cases had been reported. In early 2015, autochthonous Zika virus transmission was detected for the first time in the Americas, in Brazil (*3*). After explosive spread in the Americas, transmission has been documented in 48 countries and territories, including the United States (*4*). Most Zika virus infections result in inapparent or mild illness; symptoms include fever, rash, malaise, and conjunctivitis. However, during the outbreak in Brazil, Zika virus was associated with serious congenital outcomes, including microcephaly (*5*), ocular abnormalities (*6*), meningoencephalitis (*7*), and myelitis (*8*), and Guillain-Barré syndrome in many age groups (*2*). These complications and the rapid spread of the virus prompted the World Health Organization to declare Zika virus a public health emergency of international concern (*5*).

There are 2 primary Zika virus lineages: Asian and African (*9*). The Zika virus strain currently circulating in the Americas (American lineage) is derived from the Asian lineage (*10*). Because no vaccines or antiviral drugs are available (*11*), efforts to prevent Zika virus infection focus on controlling mosquito vectors. Historically, Zika virus has been isolated from several *Aedes* spp. mosquitoes, including multiple sylvatic species in Africa (*1**,**12**,**13*) and the domestic species *Ae. aegypti* in Malaysia (*1*) and Mexico (*14*). *Ae. aegypti* mosquitoes are the main urban vector of other medically important urban arboviruses with similar origins, such as dengue virus (DENV), chikungunya virus (CHIKV), and yellow fever virus. However, studies of *Ae. aegypti* mosquito susceptibility for Zika virus have yielded varied results; some have suggested relative refractoriness (*15*). This finding has led to speculation that other vectors common in tropical cities, such as *Ae. albopictus* mosquitoes (*16*), implicated in a Gabon epidemic (*17*), and *Culex quinquefasciatus* mosquitoes, common in tropical cities, could be Zika virus vectors (*13*).

One hypothesis for the sudden emergence of Zika virus epidemics since 2007 is viral adaptation for more efficient transmission by *Ae. aegypti* mosquitoes (*18*). A precedent for this mechanism is the adaptation of CHIKV for infecting *Ae. albopictus* mosquitoes, mediated through a series of envelope glycoprotein substitutions. This adaptation enabled the dramatic spread of CHIKV in the Indian Ocean Basin, Asia, and Europe since 2005 (*19*). Similar adaptive evolution of the Asian and/or American lineages of Zika virus for transmission by *Ae. aegypti* mosquitoes could explain the lack of past major urban outbreaks.

During 2016, we tested this hypothesis by examining the ability of 3 Zika virus strains representing African, Asian, and American lineages to be transmitted by *Ae. aegypti* mosquitoes. Because geographically disparate populations of this species can vary in their susceptibility to flaviviruses (*20*), including Zika virus (*15*), we tested populations from 3 at-risk sites in the Americas—Brazil (Salvador), the Caribbean (Dominican Republic [DR]), and the United States (Rio Grande Valley [RGV], Texas)—with Zika virus strains from Senegal (DAK AR 41525), Cambodia (FSS 13025), and a 2015 Mexico outbreak (MEX1–7) (*14*). We also estimated the extrinsic incubation period (EIP) and viral titers in mosquito saliva and characterized differences in infection and dissemination between artificial and viremic blood meals (*21*).

## Materials and Methods

### Cells

Vero cells were purchased from ATCC (Bethesda, MD, USA). Cells were maintained in Dulbecco’s modification of Eagle’s medium (DMEM) (Invitrogen, Carlsbad, CA, USA) supplemented with 5% fetal bovine serum (FBS) (Atlanta Biologicals, Flowery Branch, GA, USA) and penicillin/streptomycin (100 U/mL and 100 μg/mL respectively) (Invitrogen) in a humidified incubator at 37°C with 5% CO_2_.

### Viruses

We used the following Zika virus strains in these studies: FSS 130125 (GenBank accession no. KU955593.1), a human isolate from Cambodia isolated in Vero cells, passaged once in C6/36 before lyophilization; DAK AR 41525 (KU955591.1), an *Ae. africanus* isolate from Senegal isolated in AP61 cells and passaged once in C6/36; and MEX 1–7 (KX247632.1), isolated from *Ae. aegypti* mosquito on Vero cells with 3 additional passages. All viruses were acquired as lyophilized stocks from the World Reference Center for Emerging Viruses and Arboviruses at the University of Texas Medical Branch (Galveston, TX, USA). Viruses were cultured once in C6/36 *Ae. albopictus* cells, followed by 3 passages in Vero cells to generate stocks for mosquito feeding. All stocks were titered by focus-forming assay (FFA) and frozen at −80°C in 30% FBS before use in artificial blood meals or mouse infections.

### Mosquitoes

Colonized *Ae. aegypti* mosquitoes from Salvador (generation F2), the DR (F6), and the RGV (F4) were housed in a 27° ± 1°C incubator (a typical temperature in tropical climates) with 80% ± 10% relative humidity in cardboard cups with mesh lids, fed 10% sucrose ad libitum, and maintained at 16:8 light:dark cycle. Mosquitoes were sex-sorted 3 days posteclosion. Twenty-four hours before experiments, sucrose was replaced with water, which was withdrawn 6 h before feeding.

### Murine Infections

Four-week-old interferon type I receptor-knockout (A129) mice were infected intraperitoneally with 1 × 10^5^ focus-forming units (FFU) of Zika virus FSS 13025 diluted in phosphate-buffered saline. This model generates viremias of 10^4^–10^7^ during 1–3 days post infection (dpi) (*22*). One animal per day was randomly selected, anesthetized with 100 mg/kg of ketamine, and placed on the screened lid of cups containing sucrose-starved *Ae. aegypti* mosquitoes (Salvador). Mosquitoes were allowed to feed for 30 min, then cold-anesthetized, and fully engorged specimens were incubated. After blood feeding, mice were euthanized and exsanguinated for viremia quantification by FFA.

### Preparation of Infectious Blood Meals and Oral infection

Artificial blood meals containing Zika virus were prepared at ≈4 × 10^4^, 4 × 10^5^, or 4 × 10^6^ FFU/mL. Blood meals comprised 1% (wt/vol) sucrose, 7.5% fetal bovine serum (FBS), 12.5% washed human erythrocytes (University of Texas Medical Branch blood bank), 900 μM adenosine triphosphate, and viral dilutions in DMEM containing 2% FBS and 100 U/mL penicillin and 100 μg/mL streptomycin. After 1 h of feeding, mosquitoes were cold-anesthetized, and engorged females were extrinsically incubated. Infections were conducted in 4 separated experiments, with 1 of the 3 mosquito strains studied at a time, followed by the murine blood meal experiments.

### Mosquito Dissemination and Transmission Potential

On days 2, 4, 7, 10, and 14 after feeding, ≈9 mosquitoes per group (fewer in some groups because of feeding efficiency and survival) were cold-anesthetized, and legs were removed and placed into Eppendorf tubes containing a steel ball bearing and 500 μL of DMEM, supplemented with 2% FBS, 1% penicillin/streptomycin, and 2.5 μg/mL amphotericin B (GIBCO, Waltham MA, USA). On days 4, 7, 10, and 14, after removal of legs, mosquitoes were immobilized and their proboscis inserted for 30 min of salivation into a sterile 10-μL micropipette tip containing 8 μL of FBS, after which the expectorated saliva/FBS was added to 100 μL of DMEM. Mosquito bodies were then triturated for 5 min at 26 Hz in a Tissue Lyser II (QIAGEN, Venlo, the Netherlands) in microfuge tubes containing a steel ball bearing and 500 μL of mosquito media. On day 2 after infection, only bodies and legs were collected. For mosquitoes fed on viremic mice, samples were collected 3, 7, and 14 days after feeding. Homogenized mosquito samples were clarified by centrifugation at 200 × *g* for 5 min.

### FFA

FFAs were conducted as described previously (*23*) for viral stocks, blood meals, and all mosquito samples by inoculating 96-well plates of nearly confluent Vero cells with 50 μL of sample supplemented with 50 μL of mosquito media. After a 3-day incubation, plates were fixed, washed, and blocked before overnight incubation with mouse anti– Zika virus (strain MR-766). Plates were washed and incubated with goat anti-mouse secondary antibody conjugated to horseradish peroxidase (KPL, Gaithersburg, MD, USA). Plates were washed and developed with aminoethylcarbazole solution (Enzo Diagnostics, Farmingdale, NY, USA) prepared according to the manufacturer’s protocol for detection.

### Saliva Titration

Positive saliva samples were titrated by FFA on 24-well plates of Vero cells. Means and SDs were calculated for all positive samples. Samples that were positive during initial screening but below the limit of detection (10 FFU) for the titration assay were given a value of limit of detection–1 for calculations.

### Statistical Analysis

For mosquitoes fed on artificial blood meals, the effect of mosquito strain, virus strain, and dpi, as well as interactions among these, on percentage of bodies infected was analyzed by using a nominal logistic regression, with separate analyses for each blood meal titer (≈4, 5, or 6 log_10_ FFU/mL). Because of the large number of comparisons, the threshold for significance (α) was set to an arbitrary but conservative threshold of 0.005. Next, the effects of mosquito strain, virus strain, and dpi on dissemination, measured as the percentage of infected bodies that produced infected legs, were analyzed by using a nominal logistic regression, with separate analyses for each blood meal titer. Similarly, the effects of mosquito strain, virus strain, and dpi on transmission, measured as the percentage of mosquitoes with disseminated infection that secreted virus in the saliva, were analyzed by using a nominal logistic regression, with separate analyses for each blood meal titer. For both dissemination and saliva infection, interactions among each of the 3 independent variables were not fully explored, because some combinations were not included (i.e., some mosquito strain × virus strain × dpi combinations did not yield infected bodies). Virus titer in the saliva was not subject to statistical analysis because of small sample sizes. The effects of feeding mode (mouse versus artificial blood meal), virus titer, and dpi on the percentage of infections, dissemination, and transmission in Salvador *Ae. aegypti* mosquitoes fed on Zika virus strain FSS were analyzed by nominal logistic regression.

## Results

We detected no statistically significant interactions among mosquito strain, virus strain, and dpi in any analysis of infection, dissemination, or transmission after mosquitoes fed on an artificial blood meal (Table 1). Frequently, dpi significantly affected infection, dissemination, and transmission as expected based on the need for replication and dissemination in the mosquito, so these data are not presented in detail here.

**Figure 1:**
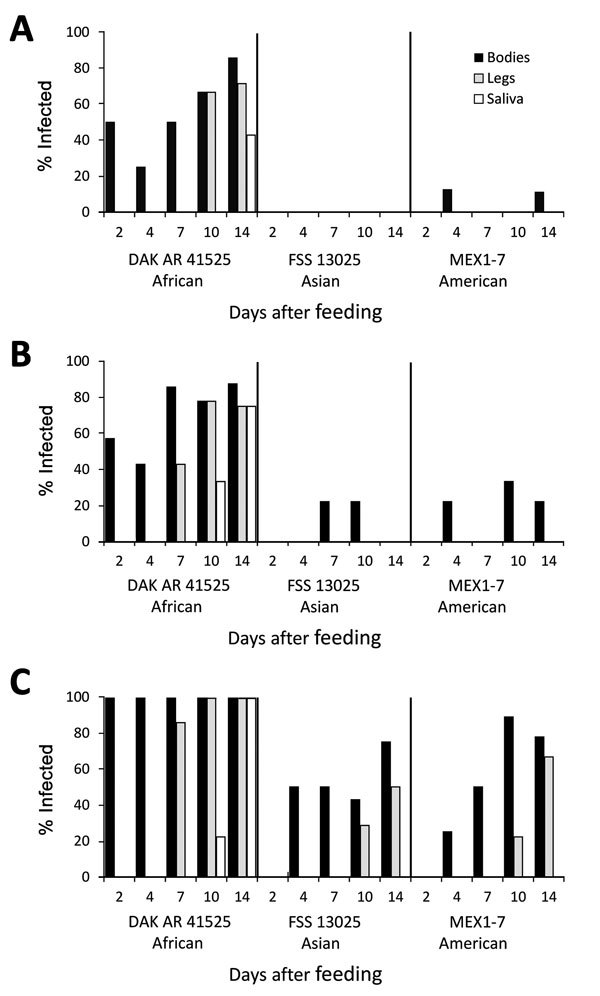
Infection, dissemination, and transmission of 3 Zika virus strains by *Aedes aegypti* mosquitoes from Salvador, Brazil, after artificial blood meals with a concentration of 4 log_10_ (A), 5 log_10_ (B), or 6 log_10_ (C) focus-forming units/mL.

**Figure 2:**
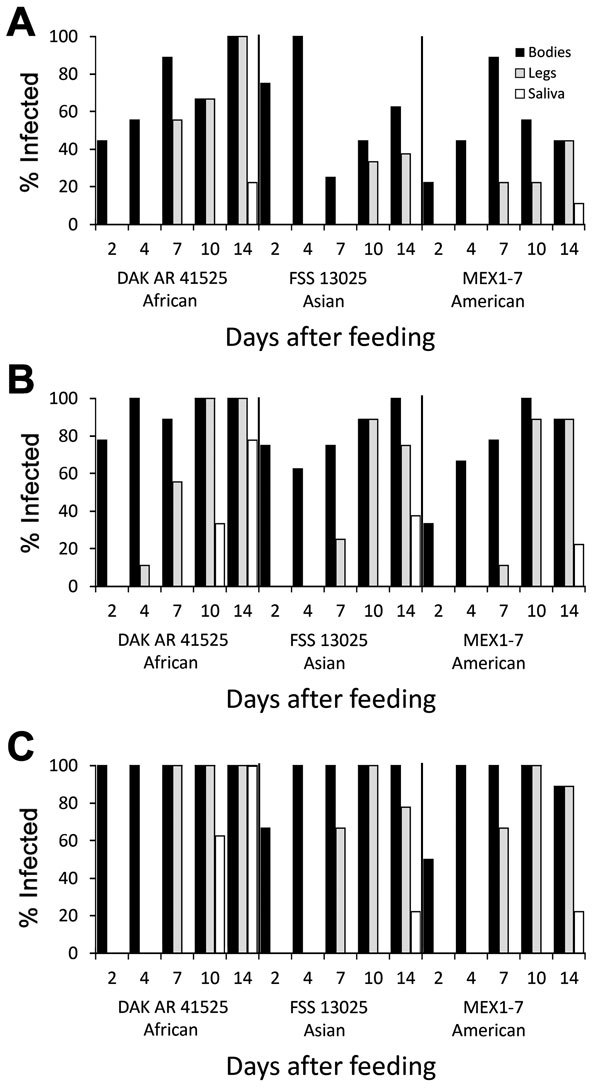
Infection, dissemination, and transmission of 3 Zika virus strains by *Aedes aegypti* mosquitoes from the Dominican Republic after artificial blood meals with a concentration of 4 log_10_ (A), 5 log_10_ (B), or 6 log_10_ (C) focus-forming units/mL.

**Figure 3:**
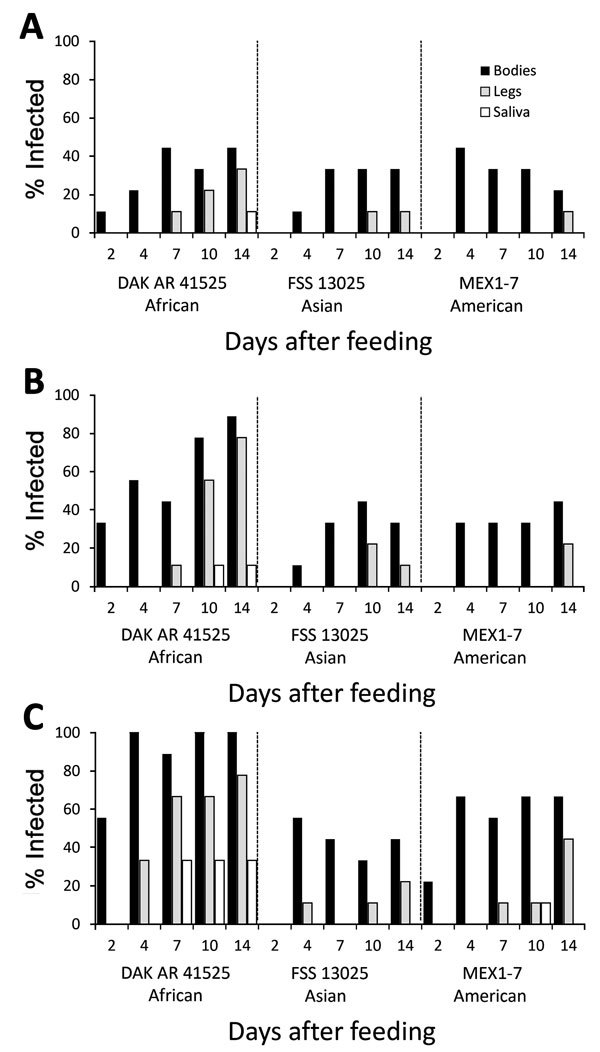
Infection, dissemination, and transmission of 3 Zika virus strains by *Aedes aegypti* mosquitoes from the Rio Grande Valley, Texas, USA, after artificial blood meals with a concentration of 4 log_10_ (A), 5 log_10_ (B), or 6 log_10_ (C) focus-forming units/mL.

When *Ae. aegypti* mosquitoes were fed on artificial blood meals at doses of 5 or 6 log_10_ FFU/mL of Zika virus, DAK AR 41525 (Figure 1, panels B, C; Figure 2, panels B, C; Figure 3, panels B, C) produced a significantly higher percentage of infection than did the same titers of strain FSS and MEX1–7 (Figure 1, panels B, C; Figure 2, panels B, C; Figure 3, panels B, C) across all 3 strains of *Ae. aegypti* (p<0.001 at 5 log_10_ FFU/mL, p<0.002 at 6 log_10_ FFU/mL). In addition, at the 2 higher doses, disseminated DAK AR 41525 infections produced a higher percentage of infectious saliva (p<0.004 at 5 log_10_ FFU/mL, p<0.0001 at 6 log_10_ FFU/mL). DAK AR 41525, however, did not result in a higher percentage of infections resulting in dissemination to the legs (a proxy for the hemocoel). At an artificial blood meal concentration of ≈4 log_10_ FFU/mL, we found no significant difference among the 3 Zika virus strains in infection, dissemination, or transmission. For all artificial blood meal concentrations, FSS 13025 and MEX 1–7 produced similar infection, dissemination, and transmission percentages in each mosquito population.

When *Ae. aegypti* mosquitoes were fed on artificial blood meals at doses of ≈4, 5, or 6 log_10_ FFU/mL of Zika virus, a significantly greater percentage of mosquitoes from the DR (Figure 2) became infected than from the RGV (Figure 3) and Salvador populations (Figure 1) (p<0.001 at 4 and 5 log_10_ FFU/mL, p<0.002 at 6 log_10_ FFU/mL). At doses of 5 and 6 log_10_ FFU/mL, a greater percentage of *Ae. aegypti* mosquitoes from the DR with disseminated infections had infectious saliva (p<0.004 at 5 log_10_ FFU/mL, p<0.0001 at 6 log_10_ FFU/mL). *Ae. aegypti* mosquitoes from the DR, however, did not have significantly higher percentages of infections that disseminated. For all artificial blood meal doses and Zika virus strains, *Ae. aegypti* from Salvador and the RGV had similar infection, dissemination, and transmission percentages.

**Figure 4:**
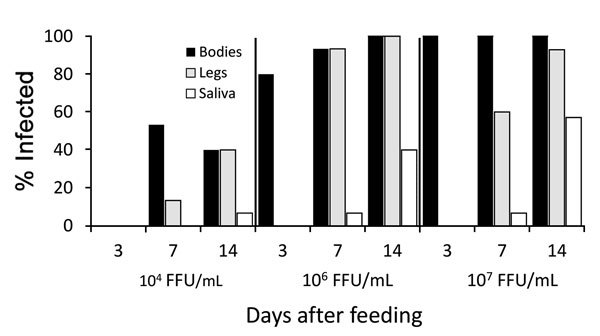
Infection, dissemination, and transmission of the Zika virus strain FSS 13025 by *Aedes aegypti* mosquitoes from Salvador, Brazil, after blood meals from infected A129 mice with viremic titers of 4 log_10_, 6 log_10_, or 7 log_10_ focus-forming units/mL.

Because virus titers and sampling days for mosquitoes fed on mice (4, 6, and 7 log_10_ FFU/mL sampled 7 and 14 days postfeeding) and artificial blood meals (4, 5, and 6 log_10_ FFU/mL sampled 2, 4, 7, 10, and 14 dpi) did not completely overlap, we first compared mosquito infection only for blood meal titers (≈4 and 6 log_10_ FFU/mL) and dpi (7, 14 dpi) that coincided between the 2 feeding methods (Figure 1, panels A [middle panel], C [middle panel]; Figure 4). A nominal logistic regression using these data (N = 81) showed no significant interactions among the independent variables; virus titer (χ^2^ 24.3, df = 1, p<0.0001) and feeding method (χ^2^ 9.7, df = 1, p<0.0019) significantly affected the likelihood of infection, whereas dpi did not (χ^2^ 0.33, df = 1, p = 0.56). Using this same dataset, we found that virus titer, feeding method, and dpi all significantly affected dissemination from infected bodies to legs (N = 50, p<0.0001 for all 3 variables). Because only 8 mosquitoes in this group produced infected saliva, we did not attempt analysis on this small sample. However, it was striking that only mosquitoes fed on mice produced infected saliva. An analysis using all data from Salvador mosquitoes fed on Zika virus strain FSS 13025 in artificial blood meals and mice revealed a significant effect of all 3 independent variables on infection (p<0.0001 for all comparisons), with infection being greater at higher blood meal titers and later time points after infection and from blood meals acquired from mice.

*Ae. aegypti* mosquitoes from Salvador exhibited a minimum EIP of 10 days after artificial infection with Zika virus strain DAK AR 41525 at 5 and 6 log_10_ FFU/mL and 14 days after infection with FSS 13025 or MEX1–7 strains at 6 log_10_ FFU/mL and DAK AR 41525 at 4 log_10_ FFU/mL. *Ae. aegypti* mosquitoes from the DR exhibited an EIP of 10 days after artificial infection with Zika virus strain DAK AR 41525 at 5 and 6 log_10_ FFU/mL and 14 days after infection with FSS 13025 at 5 or 6 log_10_ FFU/mL, MEX1–7 strains at all 3 doses, and DAK AR 41525 at 4 log_10_ FFU/mL. *Ae. aegypti* mosquitoes from the RGV did not effectively transmit FSS 13025 or MEX1–7 at any titer (only 1 positive MEX1–7 saliva sample on 10 dpi) but showed an EIP of 7 days with strain DAK AR 41525 at 6 log_10_ FFU/mL, 10 days at 5 log_10_ FFU/mL, and 14 days at 4 log_10_ FFU/mL. Mosquitoes infected through murine blood meals showed an EIP of 7 days after a 6 or 7 log_10_ FFU/mL blood meal, and 14 days after a 4 log_10_ blood meal.

## Discussion

Because no vaccine or therapeutic drugs are available, Zika virus prevention depends on controlling the mosquito vector. Although some previous studies (*15*) showed relatively low Zika virus competence in *Ae. aegypti* mosquitoes, raising questions about the role of other potential vectors, others have shown this species to be highly competent (*24*,*25*). We demonstrated that *Ae. aegypti* mosquito competency as a vector for Zika virus in the Americas varies greatly and depends on mosquito origin, Zika virus strain, and type of blood meal used. Recent studies demonstrated that preexisting DENV antibodies in Zika virus–endemic areas might enhance Zika virus infection in vitro (*26*); other studies have conversely demonstrated that monoclonal antibodies to DENV envelope neutralize Zika virus in vitro and protect immunocompromised mice from lethal infection (*27*). The role of preexisting immunity to heterologous viruses remains unclear; thus, even a moderately competent vector, such as *Ae. aegypti* mosquitoes, might be able transmit efficiently because of its highly anthropophilic behavior and ready access to homes without screening or air conditioning in much of Latin America and the Caribbean.

In agreement with previous studies (*15*), we demonstrated significant variation in competency for Zika virus transmission among *Ae. aegypti* mosquito populations from 3 different parts of the Americas. After artificial blood meals, strains FSS 13025 and MEX1–7 were refractory to transmission in all populations; we detected only 1 positive saliva sample after large oral doses. In contrast, mosquitoes from the DR were susceptible to and able to transmit all 3 Zika virus strains. A similar difference in DENV competency has been noted in comparisons of *Ae. aegypti* mosquito populations from different geographic locations (*20*). This variation could be due to genetic differences among mosquitoes or differences in microbiome, virome, or immune activation. Understanding differences in competency and underlying mechanisms could help guide new strategies to control this vector.

In addition to differences in competency among *Ae. aegypti* mosquito populations, we showed a significant difference in infectivity among Zika virus strains. DAK AR 41525 was the only strain capable of disseminating and being transmitted by all mosquito strains. Furthermore, in mosquitoes from the DR, which were susceptible to all 3 Zika virus strains, DAK AR 41525 disseminated the most rapidly and resulted in the greatest proportion of infectious saliva. This finding is surprising given that African Zika virus strains have never been associated with outbreaks involving *Ae. aegypti* mosquitoes.

Another contribution of our findings is the higher infectivity from murine blood meals than from artificial meals. Artificial blood meals are known to be less infectious than natural meals, at least in part because of the lack of coagulation and concentration of the virus adjacent to the mid-gut epithelium (*28**,**29*). Also, in the case of DENV and St. Louis encephalitis virus, frozen stocks are less infectious for *Ae. aegypti* mosquitoes than freshly harvested, cell culture–derived virus (*30*). The FSS 13025 strain of Zika virus infected only 75% of Salvador *Ae. aegypti* mosquitoes at 6 log_10_ FFU/mL by 14 dpi from an artificial blood meal, with 67% of these infections disseminating, and 0% involving the saliva. In contrast, 14 dpi after feeding on an infected mouse with a 6 log_10_ FFU/mL viremia, 100% infection occurred; 92% of these were disseminated, and 61% of disseminated infections reached the saliva. With titers as low as 4 log_10_ FFU/mL in murine blood meals, 40% of mosquitoes became infected, of which 100% were disseminated and had Zika virus detected in saliva. This dramatic difference in competency after artificial versus viremic blood meals undoubtedly contributed to the underestimation of *Ae. aegypti* mosquitoes as a Zika virus vector in previous studies (*15**,**25*).

An important determinant of vector capacity is the EIP, that is, the time before a virus can be found in the saliva of a mosquito after an infectious blood meal. The EIP for DENV varies depending on temperature and other factors, with an average of 6.5 days at 30°C and 15 days at 25°C (*31*). The EIP for CHIKV is as short as 2 days (*32*). A short EIP facilitates rapid spread, whereas a long EIP gives a larger window for mosquito death, including by human intervention. The 7-day minimum EIP we estimated after a murine blood meal, and 7–10 days after an artificial blood meal, are comparable to those of other flaviviruses in mosquitoes incubated at similar temperatures.

Another major factor in vector transmission is the amount of virus inoculated in the saliva, which can affect pathogenesis (*21*); this value is critical for determining realistic animal model doses. We found saliva titers of up to 4 log_10_ FFU per collection, with the following mean ±SD log_10_ FFU/collection for each mosquito–virus strain combination: Salvador mosquitoes, DAK AR 41525: 2.49 ±2.93; DR mosquitoes, DAK AR 41525: 2.72 ±3.26; DR mosquitoes, MEX1–7: 2.30 ±2.35; RGV mosquitoes, DAK AR: 2.20 ±1.96; Salvador mosquitoes, FSS 13025 infected through a murine blood meal: 2.77 ±3.00. Because of the dearth of positive saliva samples, no statistically significant differences were found for these means. These infectious saliva titers are based only on a small number of positive samples after artificial blood meals. Some studies have found that in vitro salivation overestimates the amount of an arbovirus inoculated in vivo (*33*); others have found the inverse (*34*). Additional studies are needed to precisely determine the amount of virus transmitted by a Zika virus–infected *Ae. aegypti* mosquito.

Ideally, in investigations of viral adaption to vectors, virus and mosquito origins should be matched. The mosquitoes to match the locations of the Zika virus strains reported here were unavailable. However, vector-adaptive mutations in arboviruses are unlikely to remain geographically isolated because they spread more efficiently (*35*,*36*). Therefore, adaptive evolution was investigated on the basis of available mosquitoes with minimal colonization histories, from sites at risk for Zika virus transmission or with reported autochthonous transmission. Surprisingly, despite the use of minimally colonized mosquitoes, most susceptible population of *Ae. aegypti* from the DR had the longest history of 6 generations. Previous studies demonstrated altered DENV-2 susceptibility for *Ae. aegypti* colonized for >4 generations (*37*).

Although human Zika virus viremia is not well characterized, a Micronesia study found viral RNA concentrations of 900–729,000 RNA copies/mL (*38*). Recent case studies have estimated ranges of 1.47–2 log_10_ PFU/mL (*39*), 0.49–3.39 log_10_ FFU/mL (*14*), 2.20–2.75 log_10_ PFU/mL, and 1.88–2.80 log_10_ PFU/mL (*40*). This wide range might reflect the sampling of most patients after peak viremia has passed, which complicates selecting realistic doses for mosquito competency studies. 
